# General practitioners’ attitudes and barriers to patient activation in cardiovascular disease prevention: insights from the DECADE study

**DOI:** 10.1186/s12875-025-02798-x

**Published:** 2025-03-28

**Authors:** Willy Gräfe, Iris Tinsel, Maja Börger, Thomas Kloppe, Andy Maun, Henna Riemenschneider

**Affiliations:** 1 Department of General Practice, Faculty of Medicine Carl Gustav Carus, Dresden University of Technology, Fetscherstraße 74, 01307 Dresden, Germany; 2 Section of Health Care Research and Rehabilitation Research, Institute of Medical Biometry and Statistics, Medical Faculty and Medical Center, University of Freiburg, Freiburg, Germany; 3 Institute of General Practice/Family Medicine, Medical Faculty and Medical Center, University of Freiburg, Freiburg, Germany; 4 Department of General Practice and Primary Care, University Medical Center Hamburg-Eppendorf, Hamburg, Germany

**Keywords:** Patient activation, Primary care, Cardiovascular diseases, Prevention, Self-management, Lifestyle counselling

## Abstract

**Introduction:**

Cardiovascular diseases (CVD) are the most common cause of death in Germany. General practitioners (GPs) have an important role in supporting patients in the prevention of CVD. The DECADE intervention was developed to encourage patients to improve self-management in order to prevent CVD, addressing both GPs and patients. This study focused on GPs attitudes towards patient activation and its relation to the level of activation on their patients, possible barriers according to lifestyle counselling and attitudes towards interprofessional consultations.

**Methods:**

Questionnaire-based cross-sectional analysis was conducted within the DECADE-cRCT. GPs attitudes to patient activation was measured by using seven items of the Clinician Support for Patient Activation Measure (CS-PAM). The degree of activation of the patients was measured by Patient Activation Measure (PAM13-D). The Barriers and attitudes towards responsibilities for lifestyle counselling were assessed using self-generated items on a 5-point Likert scale. Association between CS-PAM and PAM13-D was analysed using a linear mixed model.

**Results:**

79/82 GPs responded. Mean CS-PAM score of GPs at baseline was 23.00 (range 13–27, max. 28). GPs reported lack of time, funding and uncertainty of the impact as main barriers to the routine implementation of lifestyle counselling. GPs see themselves as primarily responsible for conducting lifestyle counselling, but they also emphasize the importance of interprofessional cooperation. No significant correlations between CS-PAM and PAM13-D were found.

**Conclusion:**

GPs perceive patient activation as important. Most GPs reported that they already provide lifestyle counselling as part of their routine practice. Overcoming the identified barriers in GPs lifestyle counselling is a prerequisite for effective and patient-centred consultation on cardiovascular risk factors. The interprofessional collaboration advocated by GPs could relieve the burden on GPs and thus reduce these barriers.

**Trial registrations:**

The DECADE-study is registered in the German Clinical Trials Register (DRKS-ID: DRKS00025401; Trial registration date: 2021/06/21) and in the International Clinical Trials Registry Platform (ICTRP): https://trialsearch.who.int/Trial2.aspx?TrialID=DRKS00025401.

**Supplementary Information:**

The online version contains supplementary material available at 10.1186/s12875-025-02798-x.

## Introduction

The most common cause of death in Germany in 2020, with a mortality rate of 34%, was cardiovascular disease (CVD) [1]. Lifestyle-related risk factors such as tobacco and alcohol consumption, an unhealthy diet, lack of physical activity and obesity are associated with premature CVD. The presence of several risk factors increases the cardiovascular risk even more [1, 2]. Preventing CVD involves supporting patients’ health-promoting behaviour. The concept of patient activation encompasses patients’ health knowledge and their ability to improve or maintain their health, including lifestyle changes [3]. GPs, very often as primary contact for health issues for the patients, play an important role in the prevention of CVD by supporting patients in the implementation of lifestyle changes. There is evidence that lifestyle interventions in primary care can reduce cardiovascular risk factors [4, 5]. Nevertheless, it is often difficult for patients to implement lifestyle changes in the long term.

Previous research has shown that lifestyle interventions with a focus on self-management or patient activation can improve patients’ knowledge about diseases (including CVD) as well as their health-related behaviours [6–11]. In detail, prioritising patient activation means that GPs support their patients in changing their lifestyle behaviours by setting behavioural goals with them, by developing realistic steps to achieve these goals and by discussing solutions to lifestyle issues of the patients. The study by Alvarez et al. [12] showed that GPs’ attitudes towards patient activation were positively associated with the actual level of activation of their patients. The study found gender differences: female GPs had a more positive attitude towards patient activation than male GPs [12]. In contrast, the study conducted by NHS England (2015) found no significant effect of age and gender on GPs’ attitudes towards patient activation [13]. Previous studies have shown that GPs in Germany consider health counselling for lifestyle changes as an essential task [14, 15]. Nevertheless, the counselling skills vary regarding the topic (i.e. physical activity, nutrition, smoking cessation, sexually transmitted infections) [14–16].

Interventions in GP practices can positively impact patients cardiovascular health [17]. GPs serve as both healthcare providers and health counsellors, engaging in shared decision-making with patients to set lifestyle goals [18]. However, GPs in Germany and beyond (i.e. Spain, Sweden, Greece, Georgia) report non-adherence, time constraints, high workload in GPs practice as well as lack of financial compensation as major barriers to the implementation of routine lifestyle counselling for reducing CVD risk [19–22].

DECADE (“Decision Aid, Action Planning, and Follow Up Support for Patients to Reduce the 10-Year Risk of Cardiovascular Diseases”) is a complex intervention, tested in a cluster-randomised controlled trial (cRCT) with three measurement times (t0 = baseline, t1 = 6 months and t2 = 12 months). The study was conducted in cooperation between the medical centres of the Universities of Freiburg, Dresden and Hamburg-Eppendorf and GP practices in the respective regions. The aim of the DECADE intervention is to enhance self-management of the patients to reduce their CVD. The cRCT is described in detail in the published study protocol from 2023 [23]. The pilot study from 2018 with 87 patients from six GP practices in the area of Freiburg, Germany, has shown positive effects of the DECADE intervention on patient activation (PAM13-D) [3, 24].

This study used the baseline dataset to explore how GPs’ attitudes toward patient’ activation relate to their patients’ actual activation levels and whether this is influenced by GPs’ gender or age. It also examined barriers to counselling and identified attitudes towards the perceived responsibility for health counselling. Addressing these barriers can enhance tailored, patient-centred CVD prevention by GPs, possibly in interprofessional collaboration.

## Methods

### Sample size

The primary outcome of the DECADE study was patient activation measured by the PAM13-D [3], which is why the sample size of the entire study was based on this outcome. The exact calculation of the sample size is explained in the study protocol [23]. No explicit sample size calculation was performed for this cross-sectional questionnaire-based analysis.

### Recruitment

The GPs were recruited by the the Institutes of General Practice/Family Medicine at the Medical Faculty and Medical Centre of the University of Freiburg, the Faculty of Medicine Carl Gustav Carus of Dresden University of Technology and the University Medical Centre Hamburg-Eppendorf in their respective regions. Each of the three participating universities (Freiburg, Hamburg, and Dresden) informs GPs in their respective regions about the DECADE study through postal and electronic mail, phone calls, in-person meetings, and online events. Additionally, the study has been mentioned in several medical journal articles. GPs who wish to participate sign an agreement with their regional institute of general practice before being randomly assigned to an intervention arm. Prior to knowing their assigned intervention arm, each GP completes an initial questionnaire. Once the study has begun, the enrolment of eligible patients begins. There were no inclusion or exclusion criteria for GPs to participate in the study. To keep the workload for the GPs manageable, each GP recruited up to 12 patients with at least one cardiovascular risk factor. The recruitment of the patients is described in more detail in the study protocol. Likewise, the DECADE-cRCT [23]. The data collection for the GP and patient questionnaires took place from November 2021 to January 2023.

### Instruments

GPs were surveyed before the beginning of the patient recruitment. Data included sociodemographic (age, gender), practice structure, experiences and attitudes towards barriers as well as perceived responsibility for lifestyle counselling and patient activation. Seven items (rated on a 5-point Likert scale) from the Clinician Support for Patient Activation Measure (CS-PAM) questionnaire [25] (that originally consists of 14 items) were selected to assess GPs’ views on patient activation (see Appendix 1). The chosen items were specifically relevant for the research question, minimized respondent burden to increase GP participation, and avoided overlap with other questionnaire items. The SDM-Q-Doc was also included in the questionnaire [26], which overlaps with the CS-PAM in terms of content. Accordingly, only those CS-PAM items were selected that do not overlap with the SDM-Q-Doc in terms of content.

The higher the CS-PAM sum score (range: 0–28), the more critical GPs considered the skills and behaviours of patient self-management. All seven items of the CS-PAM had to be answered by the GPs for the sum score to be calculated. The CS-PAM was translated by the study team in an iterative process, as no German version had previously been available.

GPs who reported limited implementation of counselling in their daily practice were asked to respond to a follow-up question regarding barriers to lifestyle counselling, using seven self-generated items rated on a 5-point Likert scale (0 = strongly disagree, 4 = strongly agree) (Appendix 2). Attitudes towards the perceived responsibility for health counselling were assessed with four additional self-developed items on a 5-point Likert scale (0 = strongly disagree, 4 = strongly agree) (Appendix 3). The items were created based on the literature. The GP questionnaire was pre-tested for feasibility and comprehensibility before the actual study. This allowed the GP-developed items and the German translation of the CS-PAM to be iteratively improved with the GPs.

Additionally, the patients included in the study (up to twelve per GP) were surveyed before the start of the DECADE intervention. The patients completed a multi-page questionnaire assessing their state of health, health behaviour, and sociodemographic data. The level of patient activation was evaluated using the German Version of the Patient Activation Measure questionnaire (PAM13-D), where items are rated on a 4-point Likert scale (0 = disagree, 3 = agree) [3]. A transformed sum score ranging from 0 (lowest activation) to 100 (highest activation) was calculated for each patient, provided that at least 50% of the items were answered.

### Statistical analysis

The data were analysed descriptively and presented as means with standard deviations or medians and range for metric variables and as absolute and relative frequencies for non-metric variables. Group-specific differences in data that were not normally distributed were tested for significance using the Mann-Whitney U test or Kruskal-Wallis-Test for independent samples. Associations between CS-PAM and age were calculated using Spearman’s Rho. The CS-PAM values of the GPs were compared with the PAM13-D values of their respective patients. To investigate whether the CS-PAM is associated with the PAM13-D and whether the age and sex of the GP play a role, a linear mixed model was calculated. The significance level was set at *p* ≤ 0.05. The analysis was limited to cases with complete CS-PAM data and at least 50% of PAM13-D items filled out. Statistical analyses were performed using IBM SPSS 28.0.

## Results

### General practitioners

The baseline questionnaire (t0) was completed by 79 out of 82 GPs before the intervention. The surveyed GPs were from the regions of Freiburg (*n* = 28), Hamburg (*n* = 26) and Dresden (*n* = 25). The respondents had a mean age of 47.7 years (SD = 10.5) and 59.5% were female (*n* = 47). More than half of the respondents (51.9%) had been working in GP care setting for 10 years or less. GPs estimated that they treated a mean of 1134 statutory health insurance patients per quarter (SD = 485), with estimates ranging from 200 to 2500. GPs’ estimates of the percentage of patients treated for cardiovascular diseases varied substantially, ranging from 5 to 90% (SD = 18.4%). More than half (*n* = 46) of all GPs stated to have participated in events, studies or similar initiatives aimed for improving doctor-patient communication or patient participation within the last 5 years.

The median sum score of the CS-PAM at baseline level was 23.00 (range: 13 to 27 points) out of a maximum of 28 points. Female GPs showed a significantly higher CS-PAM score than male GPs (24.00 vs. 22.00; Mann-Whitney U-test; *p* = 0.041), and GPs in Freiburg demonstrated a significantly higher CS-PAM score than GPs in Dresden (23.50 vs. 22.00; Kruskal-Wallis Test; *p* < 0.001). GPs who routinely conduct counselling sessions had a significantly higher CS-PAM score than GPs who do not routinely conduct counselling sessions (23.00 vs. 21.00; Mann-Whitney U-test; *p* = 0,029) (Table 1). Accordingly, the distribution of CS-PAM differs by gender, region, and whether routine counselling sessions are conducted. The age of the GPs was not associated with the CS-PAM.

**Table 1:** CS-PAM scores of gps depending on gender, location and performance of routine counselling sessions

	Sample description of GPs	Median CS-PAM score (0–28) (Range)	Statistical tests
Valid data (GPs)	*n* = 79	23.00 (13.00–27.00)	-
Gender			
Male Female	32 (40.5%) 47 (59.5%)	22.00 (13.00–27.00) 24.00 (17.00–27.00)	Mann-Whitney U U = 955.00; z = 2.041 *p* = 0.041
Location			
Freiburg Hamburg Dresden	28 (35.4%) 26 (32.9%) 25 (31.7%)	23.50 (19.00–27.00) 23.00 (17.00–26.00) 22.00 (13.00–27.00)	Kruskal-Wallis H = 2.761 *p* = 0.251
Routine counselling sessions			
Yes No	64 (81.0%) 15 (19.0%)	23.00 (18.00–26.00) 21.00 (13.00–27.00)	Mann-Whitney U U = 653.00; z = 2.177 *p* = 0.029

**Fig. 1:**
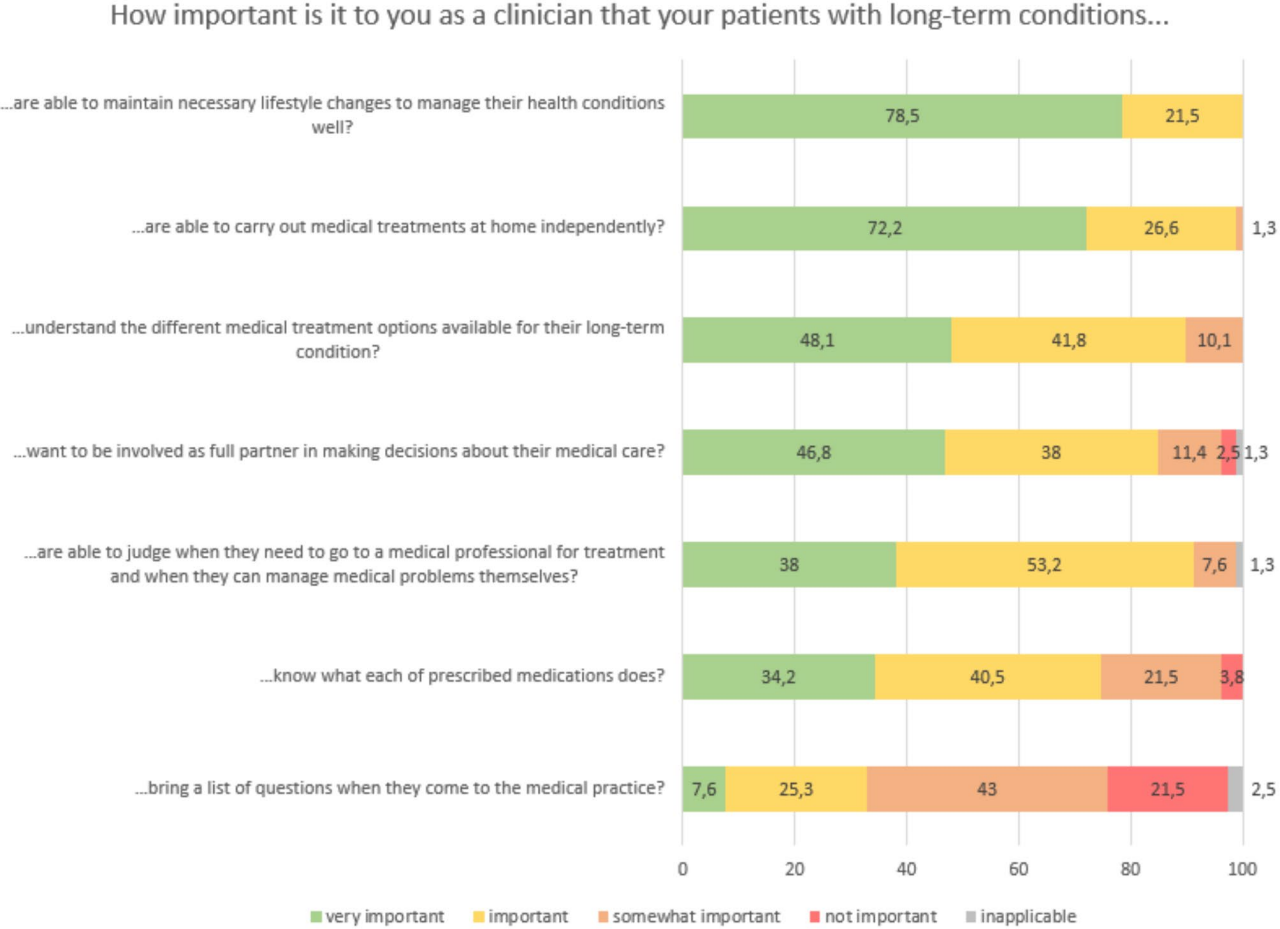
GPs evaluation towards the importance of individual items for patient activation (CS-PAM) in per cent

Analysis of answers to individual CS-PAM items showed that GPs prioritized two aspects: patients’ ability to implement and sustain lifestyle changes, and their ability to follow recommended medical treatments at home. In contrast, patients bringing a list of questions to the consultation was rated as the least important aspect by participating GPs (Fig. 1).

The five response categories of the 5-point Likert scale for assessing the barriers to counselling were aggregated into three categories. Responses of “strongly agree” and “agree” were summarised as “agree”. “Partly agree” remained unchanged, while “rather disagree” and “strongly disagree” were grouped together as “disagree”. The reason for this was that in some response categories the n was very small. For participating GPs who had stated to either only partially implement or to not implement at all detailed counselling sessions in their everyday practice (*n* = 15 (19%), female = 9; male = 6), three frequently selected barriers were identified. The first barrier was uncertainty regarding the impact of the intervention (86.6%, *n* = 13), the second a lack of time (80%, *n* = 12), and the third a lack of financial compensation (73.3%, *n* = 11). The statement “if I had better skills in counselling” received the least approval to be a relevant barrier (40%, *n* = 6).

**Fig. 2:**
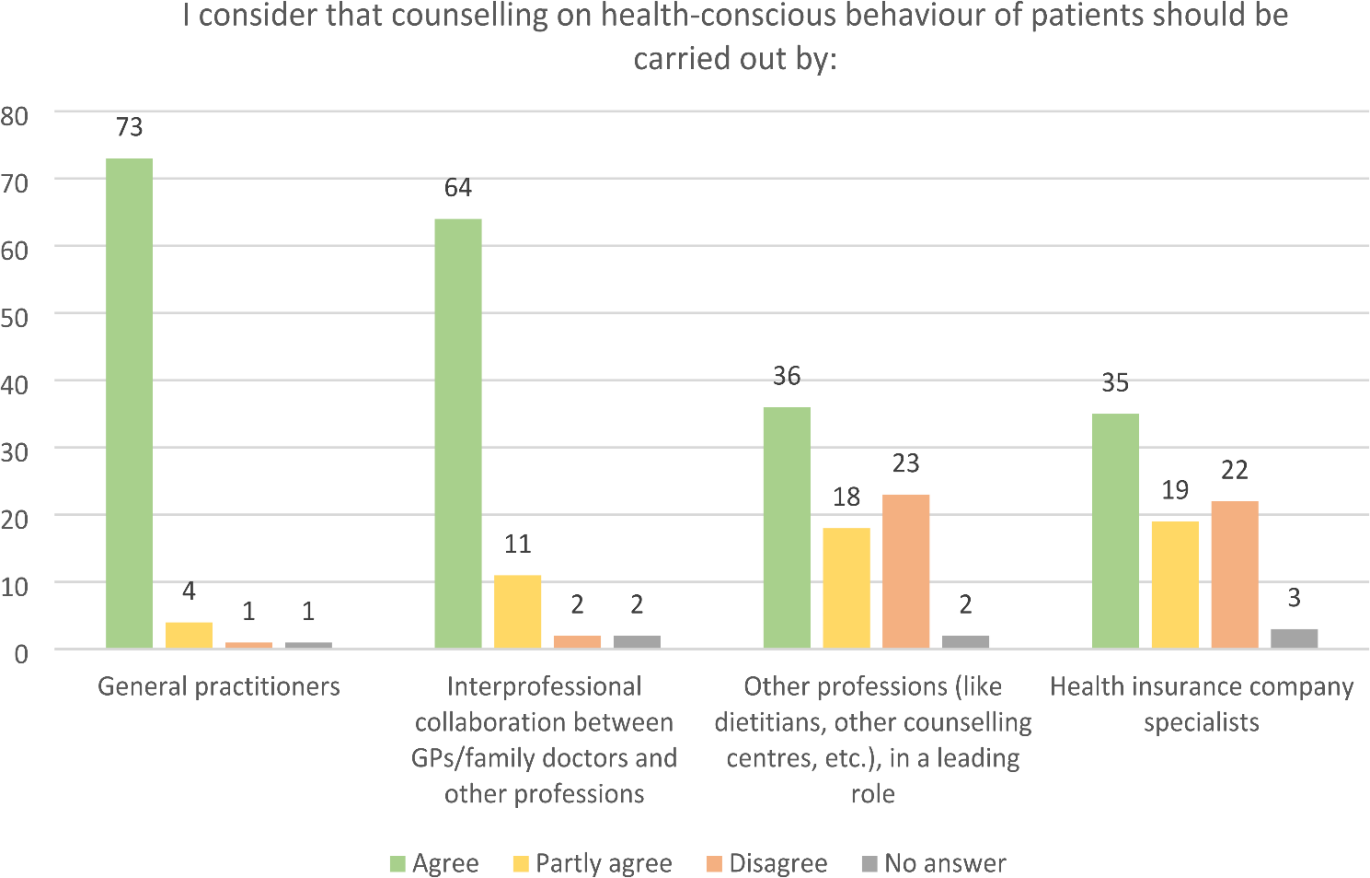
Attitudes of GPs towards responsibility for counselling on health behaviour (multiple answers possible)

The response categories of the 5-point Likert scale used to assess responsibility for counselling sessions were also aggregated into three categories. The majority of GPs (92.4%, *n* = 73) felt personally responsible for counselling sessions on health-conscious behaviour. 81.1% (*n* = 64) considered that consultations on health-conscious behaviour should involve interprofessional collaboration between GPs and other professions (Fig. 2).

### Patients

A total of 791 patients (female = 422; male = 359; no answer = 10) were recruited by 76 GPs. The mean age of the patients was 56.4 years (SD = 10.4). Further characteristics of the patients are shown in Table 2.

772 patients responded to at least 50% of the PAM13-D items and were included in the analysis. The mean PAM13-D score of the patients was 83.39 (SD = 9.18, range: 27.37 to 100.00) out of a possible 100 points. Patients (*n* = 136) under the care of GPs (*n* = 15) who do not routinely provide counselling sessions on health-conscious behaviour showed no difference in their PAM13-D score compared to patients whose GPs do offer such counselling (M = 83.45 vs. M = 83.38).

**Table 2:** Sociodemographic characteristics of the patients

Variable	*n* (%)
Total	791
Gender	
Male Female Missing	359 (45.4) 422 (53.4) 10 (1.3)
Mean age in years (SD)	56.4 (10.4)
Net monthly household income in euros:	
under 1500 1500 to under 3000 euros 3000 to under 4500 euros 4500 and more Missing	84 (10.6) 232 (29.3) 165 (20.9) 163 (20.6) 147 (18.6)
Location	
Freiburg Hamburg Dresden	296 (37.4) 234 (29.6) 261 (33.0)

### Factors influencing PAM13-D

No significant associations were found between GPs CS-PAM and patient’s PAM13-D or between the age or gender of GPs and patients’ PAM13-D. Therefore, the characteristics of the GPs could not be identified as an influencing factor on the PAM13-D of the patients.

## Discussion

This study analysed whether GPs’ attitudes towards patient activation, their age or gender are associated to the actual level of activation among their patients. It additionally investigated which barriers GPs perceive relevant to the routine implementation of cardiovascular risk counselling and whether other professions should provide such counselling. The median CS-PAM score of the GPs was 23.00 out of a possible 28 points and was in all groups (Table 1) at a high initial value. The median transformed CS-PAM (82.14; on a scale of 0-100) is higher than in comparable studies (M = 69 [25]; M = 66.1 [12]; M = 72.4 [13]; M = 80.50 to 82.11 [27]), indicating that the majority of GPs consider patient activation to be an important topic.

The patients surveyed had a mean PAM13-D of 83.39 out of possible 100 points. The PAM13-D is higher in this study than in other publications (M = 68.3 [3]; M = 66.4 [8] M = 66.08 to 67.7 [27]) but lower than in the pilot study (M = 88 [23]). The high PAM13-D and the CS-PAM found in this sample could be related to the circumstance that the majority of GPs state to already routinely provide counselling sessions on health-conscious behaviour and hence encourage the activation of their patients. However, this explanation is contradicted by the fact that no association between CS-PAM and PAM13-D was found in this study. Only 15 GPs stated that they did not routinely carry out these consultations. These GPs had a significantly lower, but still high median CS-PAM score but the PAM13-D of their patients did not differ compared to patients of the other GPs (M = 83.45 vs. M = 83.38). An explanation could be the social desirability bias, which may have influenced GPs’ responses to the CS-PAM items. Given the focus of the DECADE study on CVD prevention and patient activation, participating GPs may have felt an implicit pressure to report more positive attitudes toward patient activation, even if their actual behaviour in practice might not fully align with these values.

The results show that female GPs tend to have a more positive attitude towards patient activation compared to male GPs, which is consistent with findings from Alvarez et al. [12] who also reported significant gender differences. In addition, Alvarez et al. [12] found a significant association between GPs’ attitudes towards patient activation and the level of activation observed in their patients. No such association was identified in this study. Possible explanations for the lack of association could involve the use of only seven out of 14 CS-PAM items or the initially high level of PAM13-D scores. Moreover, almost 90% of patients had a PAM13-D score above 72.5 points (highest level of activation, level 4 [28]) and over 80% of GPs had a CS-PAM score above 20 points, which indicates little variance and may explain the lack of association.

15 GPs were asked about perceived barriers to implementing lifestyle counselling in GP practices. The lack of financial compensation and time limitations, as well as the uncertainty regarding the impact of counselling were identified as main barriers. These barriers may lead to less lifestyle counselling being provided in GP practices. Similar barriers have been reported in other studies [19–22] suggesting a broad context of challenges faced by GPs in implementing activating measures in patient care despite acknowledging the importance of patient activation. Addressing barriers to health counselling for enhancing patient activation should be a priority in health policy discussions. Ultimately, Greene et al. (2015) have shown that promoting patient activation can lead to improved health outcomes for patients and reduce costs within the healthcare system [29].

An additional strategy to reduce barriers can be derived from GPs’ responses regarding the perceived responsibility for lifestyle counselling. 92.4% of GPs see themselves as responsible for lifestyle counselling. Interestingly, of the 15 GPs who do not routinely offer counselling, 13 still believe that they are responsible for it. This indicates that these GPs in the moral dilemma of wanting to provide more counselling, but are unable to do so due to the barriers mentioned. Over 80% of GPs agreed that lifestyle counselling should involve interprofessional collaboration between GPs and other professions. Collaboration with other professions provides an opportunity to reduce the workload on GPs. For example, GPs could focus on discussing health promotion strategies and setting health goals with patients, while the actual implementation of the measures and achievement of goals could be delegated to other professionals, such as nutritionists and physical activity counsellors. Previous research has already demonstrated that interprofessional collaboration in primary care can yield positive effects on patient health [30].

A limitation of this study is that participation in the DECADE cRCT may have been influenced by a pre-existing interest in the topic of cardiovascular prevention and patient activation, which could lead to a bias in the results. A selection bias can therefore not be ruled out. Furthermore, the use of a filter question meant that only 15 GPs provided information on the barriers to lifestyle counselling. The informative value is therefore severely limited, but the results are consistent with those from other studies. Overall, this study is a cross-sectional study. The GPs’ attitudes towards patient activation therefore only relate to a specific point in time and a specific sample. The generalisability of the results is partly limited, because no full census of all GPs in Germany (*n* = 34,500) [31] was conducted, but the multicentred nature of the survey covering different regions of Germany increase the generalisability of the results. Furthermore, there was no German version of the CS-PAM, which is why the study team translated it themselves in an iterative process. However, no validation study could be carried out in advance. Further research should examine the comprehensibility, reliability and construct validity of the translated items, but the items were tested as pre-test with a small number of GPs prior to the study. Although these are limitations of the study, the present study provides insights into the attitudes and challenges of GPs with regard to patient activation.

## Conclusion

This study highlights the importance of patient activation in CVD prevention and the role of GPs in supporting patients’ self-management. While most GPs reported engaging in lifestyle counselling, they identified significant barriers such as time constraints, lack of financial compensation, and uncertainty about the effectiveness of counselling. Additionally, although GPs with higher support for patient activation were expected to have more activated patients, no significant association between GP attitudes and patient activation levels was found. The findings suggest that overcoming structural barriers and fostering interprofessional collaboration could enhance the implementation of patient-centred lifestyle counselling. Addressing these challenges through targeted policy measures may improve the effectiveness of patient activation strategies.

## Electronic supplementary material

Below is the link to the electronic supplementary material.

Supplementary Material 1

## Data Availability

Data can be obtained upon reasonable request from the authors, provided that data privacy regulations in Germany are upheld.
