# Supplementary material for: General practitioners’ attitudes and barriers to patient activation in cardiovascular disease prevention: insights from the DECADE study
Source: BMC Prim Care. 2025 Mar 28;26:86. doi: 10.1186/s12875-025-02798-x (PMC11951579; doi:10.1186/s12875-025-02798-x)
Supplement: Supplementary file 1 — Supplementary Material 1 [file 12875_2025_2798_MOESM1_ESM.docx]

**Appendix 1 – 7 Items of the CS-PAM**

How important is it to you as a clinician that your patients with long-term conditions

|  | very important | important | somewhat important | not important | inapplicable |
| --- | --- | --- | --- | --- | --- |
| …are able to maintain necessary lifestyle changes to manage their health conditions well? |  |  |  |  |  |
| …are able to carry out medical treatments at home independently? |  |  |  |  |  |
| …know what each of the prescribed medications do? |  |  |  |  |  |
| …bring a list of questions when they come to the medical practice? |  |  |  |  |  |
| …are able to judge when they need to go to a medical professional for treatment and when they can manage medical problems themselves? |  |  |  |  |  |
| …want to be involved as full partner in making decisions about their medical care? |  |  |  |  |  |
| …understand the different medical treatment options available for their long-term condition? |  |  |  |  |  |

**Appendix 2 - Barriers to lifestyle counselling**

I would give advice on health-conscious behaviour in everyday practice if

|  | fully agree | tend to agree | partly agree | tend to disagree | fully disagree |
| --- | --- | --- | --- | --- | --- |
| ...this expenditure would be reimbursed accordingly by the health insurance companies on an extra-budgetary basis |  |  |  |  |  |
| ...I would have more time |  |  |  |  |  |
| ...I would have better skills in consulting |  |  |  |  |  |
| ...I could recognize that the patient consultations bring a health benefit |  |  |  |  |  |
| ...patients would be more satisfied overall with the medical consultation |  |  |  |  |  |
| ...I would be more satisfied with the advice myself |  |  |  |  |  |
| ...I could recognize that I save time in the long term through the consultations |  |  |  |  |  |

**Appendix 3 - Responsibility for lifestyle counselling**

I am of the opinion that consultations on health-conscious behaviour of patients should be carried out by:

|  | fully agree | tend to agree | partly agree | tend to disagree | fully disagree |
| --- | --- | --- | --- | --- | --- |
| ...GPs |  |  |  |  |  |
| ... Specialist staff from the health insurance companies |  |  |  |  |  |
| ... other professions (e.g. nutritionists, other counseling centers) in a leading role |  |  |  |  |  |
| ...interprofessional cooperation between GPs and other professions |  |  |  |  |  |
